# Inter-observer variability in scoring of white matter injury on brain magnetic resonance imaging in moderate-to-late preterm infants

**DOI:** 10.1007/s00247-025-06297-0

**Published:** 2025-06-20

**Authors:** Kyle Grabowski, Liam Olsen, Greg Gamble, David Perry, Jane Harding

**Affiliations:** 1 North Shore Hospital, Auckland, New Zealand; 2 Liggins Institute, University of Auckland, 85 Park Rd, Grafton, Auckland 1142, Auckland, 92019 New Zealand; 3 Starship Children’s Health, Auckland, New Zealand

**Keywords:** Brain injury, Infant, premature, Magnetic resonance imaging, Observer variation, Reproducibility of results

## Abstract

**Background:**

Punctate white matter injury on brain magnetic resonance imaging (MRI) is described in very preterm infants (< 32 weeks’ gestation) and is predictive of poorer developmental outcomes. The reliability of scoring and the incidence and evolution of white matter injury in moderate-late preterm infants is unknown.

**Objective:**

To assess inter-observer variability in white matter injury using a published scoring system (UCSF system), and to describe changes over time in moderate-late preterm infants.

**Materials and methods:**

Infants born between 32 + 0 and 36 + 6 weeks’ gestation in the Auckland region underwent MRI scans as soon as clinically feasible after birth and again at term-equivalent age. De-identified scans were scored independently by two observers. White matter injury was graded as minimal (< 3 lesions measuring < 2 mm), moderate (> 3 lesions or lesions > 2 mm), or severe (> 5% hemispheric involvement). Scores were compared between reviewers using weighted and unweighted kappa statistics interpreted using Cohen’s criteria. Incidences were compared between scans using generalised estimating equations.

**Results:**

Scans of 101 infants were assessed. Inter-observer agreement was near perfect for the presence of white matter injury (*k* = 0.88 and 0.81 for the first and second scan respectively), and for the severity of white matter injury was near perfect at the first scan (*k* = 0.85) and substantial at the second scan (*k* = 0.80). The incidence of white matter injury detected by the two observers decreased between the first and second scans (30% to 22% and 29% to 19%), and severity also decreased.

**Conclusions:**

This scoring system can be reliably applied in moderate-late preterm infants. White matter injury is common in moderate-late preterm infants but may be underestimated when MRI is performed close to term-equivalent age.

**Graphical Abstract:**

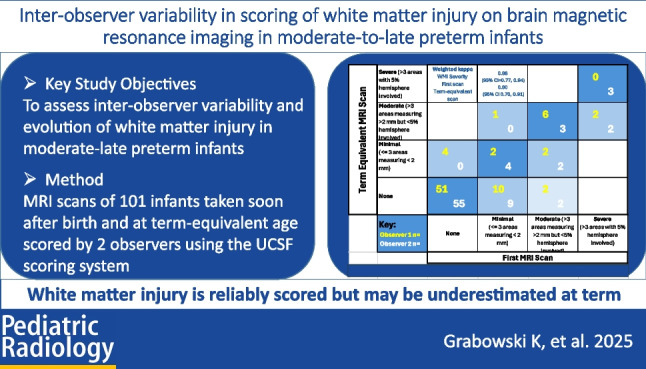

## Introduction

Moderate-to-late preterm infants born between 32 and 36 weeks’ gestation account for over 80% of all preterm births [1]. Whilst survival rates for these infants are excellent, they are 36% more likely to experience developmental delay or disability than children born at term, 50% more likely to have special education needs [2], and account for 17% of children with cerebral palsy [3]. Furthermore, moderate-late preterm infants account for almost ten times as many children with neurodevelopmental impairment as do extremely preterm infants [4], highlighting the substantial impact moderate-late preterm infants have on the healthcare burden associated with preterm birth.

Despite the global impact of moderate-late preterm birth, the reason for their increased risk of developmental impairment is unknown. Distinct patterns of punctate white matter injury are predictive of poorer developmental outcomes in very preterm infants (< 32 weeks’ gestation), with prior studies demonstrating associations with poor motor outcomes at 18 months [5, 6] and at 4 years [7]. These associations hold true even if lesions detected by magnetic resonance imaging (MRI) in the first weeks after birth are no longer visible at term-equivalent age [7].

The incidence and clinical relevance of white matter injury in moderate-late preterm infants, however, are unknown. Moderate-late preterm infants are not routinely scanned for clinical reasons, and the only research reports in this group have been of MRI at term-equivalent age [8–12], when the incidence of punctate white matter injury is 2–15% and has not been linked to later outcomes [10, 11]. However, white matter injury may be less easily detected on later scans, as the focal abnormal signal intensity is replaced by volume loss, rendering lesions less apparent. This suggests the presence of white matter injury in moderate-late preterm infants may be under-reported on scans done around term, but the extent of any under-detection is not known.

Further investigation of the extent and clinical significance of white matter injury in moderate-late preterm infants will require a way to describe white matter injury consistently. A published scoring system to evaluate the severity of white matter injury based on the number and size of lesions that was developed for very preterm infants is easy to apply clinically [5]. However, measurements of smaller foci can vary, especially on clinical radiology workstations such as IMPAX (AGFA HealthCare, Mortsel, Belgium) which confine cursor placement to voxel margins, and assessment of the percentage of hemispheric involvement is somewhat subjective.

We therefore aimed to assess inter-observer variability using this scoring system when evaluating white matter injury in moderate-late preterm infants [5]. We also aimed to assess any change in the observed incidence of white matter injury between brain MRIs undertaken soon after birth and again at term-equivalent age.

## Materials and methods

Infants born between 32 + 0 and 36 + 6 weeks’ gestation in the Auckland region were recruited either to a randomised trial of neonatal nutrition (DIAMOND) or to a prospective cohort study (MoPED) [13].

The Different Approaches to Moderate and Late Preterm Nutrition Trial (DIAMOND, ACTRN12616001199404) [13] was a randomised trial addressing questions about how to improve early nutrition in moderate-late preterm infants. Within DIAMOND, brain MRI scans are being used to assess the effects of the randomised nutritional interventions on brain growth (MR DIAMOND).

The MoPED Study (Moderate-to-late Preterm Babies’ Early Brain Development) is a prospective observational cohort study of moderate-late preterm infants to determine the incidence and clinical correlates of white matter injury, and their relationships with early brain growth and developmental outcomes.

In both studies, infants with a congenital abnormality likely to affect brain maturation or neurodevelopmental outcome or with contraindication to MRI were excluded.

Both studies received Ethics Committee approval (DIAMOND: Northern Health and Disability Ethics Committee 16/NTA/90; MoPED: Southern Health and Disability Ethics Committee 20/STH/85), and parents gave written informed consent.

The MRI protocols were identical for both studies. MRI scans were performed as soon as clinically feasible after birth (clinically stable, not on mechanical ventilation, responsible clinician considered the baby stable enough for MRI scan, visit 1) and again at term-equivalent age (visit 2).

MRI scans were performed without sedation on a 3-tesla (T) Siemens Skyra scanner (Siemens Healthineers, Forchheim, Germany). Infants were scanned after feeding and swaddling, on a vacuum mattress to reduce motion. MRI sequences included T1- and T2-weighted 3D MR images (3D T1-weighted image [TR/TE = 6/2.64 ms; 9° flip angle; FOV = 192 mm; resolution = 1.0 × 1.0 × 1.0 mm^3^] and 3D T2-weighted images [TR/TE = 3,200/571 ms; T2 variable flip angle FOV = 192 mm; resolution = 1.0 × 1.0 × 1.0 mm^3^]). Diffusion tensor imaging, susceptibility-weighted imaging, functional connectivity, and spectroscopy sequences were also collected but not used for this analysis.

**Fig. 1:**
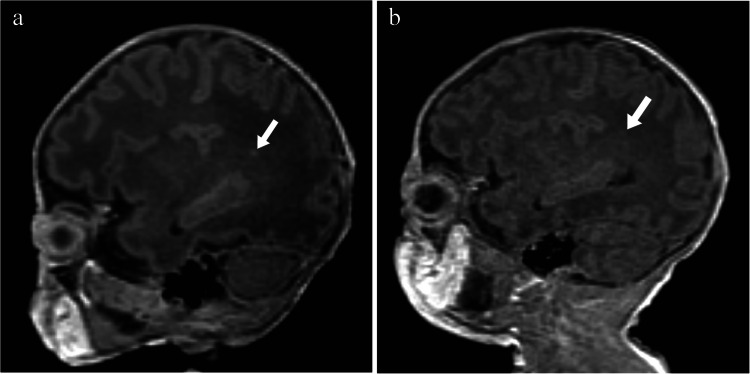
Parasagittal T1 magnetic resonance images without contrast of a boy born at 35 + 2 weeks’ gestation. **a** The image obtained on day 11 (36 + 6 weeks) shows a single area of punctate hyperintensity on the parasagittal T1 image indicating mild white matter injury (*arrow*). **b** On the image obtained on day 40 (40 + 6 weeks), the lesion is no longer visible (*arrow*)

**Fig. 2:**
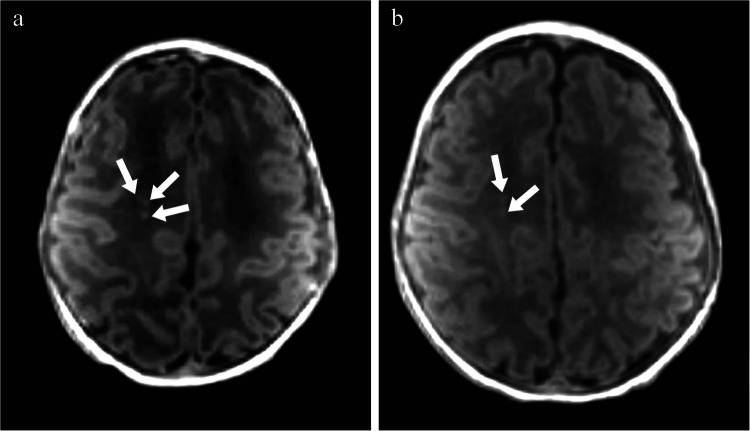
Axial T1 magnetic resonance images without contrast of a girl born at 35 + 6 weeks. **a** The image obtained on day 7 (36 + 6 weeks) shows more than three areas of hyperintensity, some > 2 mm, indicating moderate white matter injury (*arrows*). **b** The image obtained on day 31 (40 + 2 weeks) shows decreased intensity and size of the largest two lesions and apparent resolution of the third (*arrows*)

**Fig. 3:**
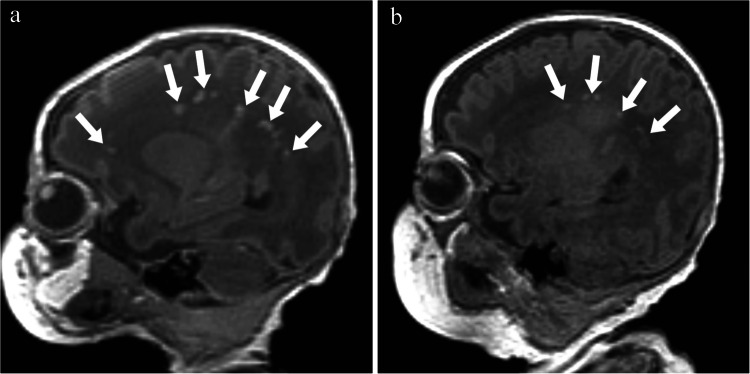
Parasagittal T1 magnetic resonance images without contrast of a boy born at 32 + 6 weeks. **a** The image obtained on day 9 (34 + 1 weeks) shows multiple areas of hyperintensity involving > 5% of the hemisphere, indicating severe white matter injury (*arrows*). **b** The image obtained on day 49 at 39 + 6 weeks shows decreased intensity and size of most lesions, with apparent resolution of some (*arrows*)

Two observers (K.G. and L.O.) at the end of their specialist radiology training underwent a single initial calibration training session with an experienced paediatric radiologist (D.P.). The observers then independently scored the de-identified scans, unaware of participant clinical history, gestational, or postnatal age at the time of the scan. A published scoring system was used to grade white matter injury as none, minimal (< 3 lesions measuring < 2 mm), moderate (> 3 lesions or lesions > 2 mm), or severe (> 5% hemispheric involvement) [5] (Figs. 1, 2 and 3).

Inter-observer variability was assessed using weighted and unweighted kappa statistics interpreted using Cohen’s criteria, whereby values below 0.2 indicate a small effect size (or slight agreement), values around 0.5 indicate a medium effect size (or moderate agreement) and values above 0.8 indicate a large effect size (or near-perfect agreement) [14].

The incidence of any white matter injury was compared for each reviewer between the first and second scans by fitting generalised estimating equations to adjust for clustering of repeat scans of the same participant (Poisson distribution and identity link function Proc Genmod, SAS v 9.4, Cary, NC). Proportions different with 95% confidence intervals (CI) for paired data were calculated for each observer.

## Results

A total of 181 scans of 101 infants (59 recruited to MR DIAMOND and 42 to MoPED) were included (100 at visit 1 and 81 at visit 2). Infants were born at a mean (SD) gestation of 33.8 (1.1) weeks and were 35.4 (1.1) weeks’ postmenstrual age at visit 1 and 40.1 (1.3) weeks at visit 2 (Table 1). Thirty-two percent of infants were female. Reasons for preterm birth included preterm prolonged rupture of the membranes (29%) and preterm labour (30%). Apgar scores < 8 at 5 min were reported in 17% (Table 1). Only four infants had possible or probable postnatal sepsis (three early, one late onset), none had chronic lung disease of prematurity, and none was screened for retinopathy of prematurity.

**Table 1:** Patient characteristics

	**Visit 1 (*n* = 100 infants)**	**Visit 2** **(*n* = 81 infants)**	**Total** **(*n* = 101 infants)**
Maternal age (years)	33.3 (6.9)	33.4 (6.6)	33.2 (6.8)
Reason for preterm birth			
Placenta praevia	3 (3%)	3 (4%)	3 (3%)
Placenta abruption	6 (6%)	4 (5%)	6 (6%)
Indeterminant APH	2 (2%)	1 (1%)	2 (2%)
PPROM	28 (28%)	26 (32%)	29 (29%)
Preterm labour	30 (30%)	22 (27%)	30 (30%)
Fetal growth restriction	1 (1%)	1 (1%)	1 (1%)
Pre-eclampsia	12 (12%)	8 (10%)	12 (12%)
Chorioamnionitis	3 (3%)	3 (4%)	3 (3%)
Other	15 (15%)	13 (16%)	15(15%)
Maternal ethnicity			
- Māori	11 (11%)	8 (9.9%)	11 (11%)
- European	42 (42%)	37 (46%)	43 (43%)
- Pacific Islander	12 (12%)	7 (8.6%)	12 (12%)
- Asian	29 (29%)	24 (30%)	29 (29%)
- Other	6 (6%)	5 (6%)	6 (6%)
Infant			
Gestational age	33.9 (1.1)	33.9 (1.1)	33.9 (1.1)
Post-menstrual age	35.4 (1.1)	40.1 (1.3)	35.4 (1.1)
Apgar < 8 at 5 min	17 (17%)	13 (16%)	17 (17%)
Female	32 (32%)	26 (32%)	32 (32%)

*APH* antepartum haemorrhage, *PPROM* preterm premature rupture of membranes.

At visit 1, there was near-perfect inter-observer agreement on the presence of any white matter injury (kappa 0.88, 95% confidence intervals (CI) 0.78, 0.98), and also on the severity of white matter injury (weighted kappa 0.85, 95% CI 0.77, 0.94) (Table 3). At visit 2, there was near-perfect inter-observer agreement on the presence of any white matter injury (kappa 0.81, 95% CI 0.65, 0.97), and substantial agreement on the severity of white matter injury (weighted kappa 0.80, 95% CI 0.70, 0.91) (Table 2).

**Table 2:** Inter-observer agreement on presence and severity of white matter injury

Visit	Presence/severity	Statistic	Estimate	Standard error	95% confidence limits
1	Presence	Simple kappa	0.88	0.05	0.78–0.98
2	Presence	Simple kappa	0.81	0.08	0.65–0.97
1	Severity	Simple kappa	0.79	0.06	0.67–0.90
1	Severity	Weighted kappa	0.85	0.04	0.77–0.94
2	Severity	Simple kappa	0.68	0.09	0.51–0.85
2	Severity	Weighted kappa	0.80	0.05	0.70–0.91

The proportion of scans with any white matter injury detected by the two observers decreased from 29/100 (29%) and 30/100 (30%) at the first scan to 15/81 (19%) and 18/81 (22%) at the second scan, absolute decreases of 11% (95% CI −23 to 2) and 8% (−21 to 5) respectively (Table 3).

**Table 3:** Severity of white matter injury recorded by each observer at each visit

	Visit 1 (*n* = 100)			Visit 2 (*n* = 81)		
	Observer 1	Observer 2	Total	Observer 1	Observer 2	Total
None	70	71	141	63	66	129
Any	30	29	59	18	15	33
- Mild	15	14	29	8	6	14
- Moderate	13	9	22	10	6	16
- Severe	2	6	8	0	3	3

At visit 1, the white matter injury was scored as mild in 15%, moderate in 11%, and severe in 4%. At visit 2, the proportion scored as mild white matter injury had decreased to 9% but there was little change in the proportion scored as moderate (10%) and severe (2%) (Table 3).

**Fig. 4:**
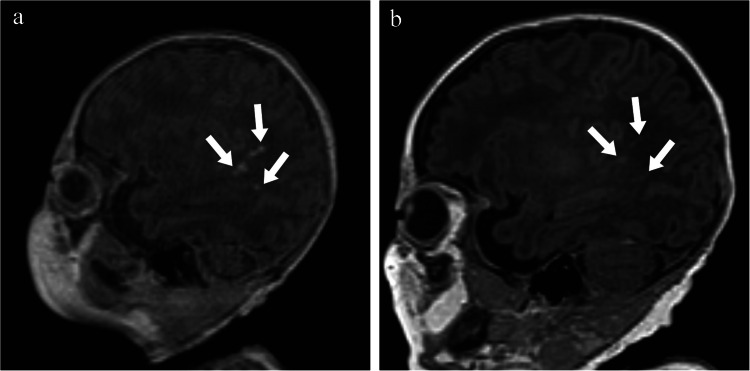
T1 parasagittal magnetic resonance images without contrast of a boy born at 35 + 6 weeks. **a** The image obtained on day 8 (37 + 0 weeks) shows multiple areas of hyperintensity indicating severe white matter injury (*arrows*). **b** The image obtained on day 45 (41 + 2 weeks) shows decreased intensity and size of all lesions (*arrows*)

However, examination of paired data from the same infants (*n* = 80) showed that 57% of scans scored as severe white matter injury at visit 1 were scored as moderate at visit 2, but none as mild or none (Table 4). Of the scans scored as moderate white matter injury at visit 1, 24% were scored as mild and 24% as no white matter injury at visit 2. Similarly, 73% of scans scored as mild white matter injury at visit 1 were scored as no white matter injury at visit 2 (Fig. 4). One observer reported new white matter injury at visit 2 in four scans (all mild), and one with more severe white matter injury at visit 2 (mild at visit 1, moderate at visit 2) (Table 4). The other observer did not report any scans with new or more severe white matter injury.

**Table 4:** Changes in severity of white matter injury between visit 1 and visit 2 recorded by each observer (*n* = 80)

Visit 2	Visit 1							
	Observer 1				Observer 2			
	None	Mild	Moderate	Severe	None	Mild	Moderate	Severe
None	51	10	2	0	55	9	2	0
Mild	4	2	2	0	0	4	2	0
Moderate	0	1	6	2	0	0	3	2
Severe	0	0	0	0	0	0	0	3

## Discussion

We aimed to assess inter-observer variability in evaluating white matter injury on brain MRI in moderate-late preterm infants using a published scoring system [5]. We found high agreement between observers regarding the presence and severity of white matter injury in moderate-late preterm infants at different ages using the published UCSF scoring system [5]. This scoring system, based on the number and size of lesions, is easy to apply clinically and has shown promising results in assessing white matter injury severity in very preterm infants [5]. The excellent inter-observer agreement observed in this study suggests that the scoring system can also be reliably applied to moderate-late preterm infants.

Most current knowledge of white matter injury in the preterm brain is obtained from research conducted in very preterm infants (< 32 weeks), where the incidence is reported to be up to 50% [15]. We found that white matter injury is also common in moderate-late preterm infants, being detectable in 30% of this cohort soon after birth. This is much higher than reported in a systematic review of 24 studies that found the incidence of white matter injury ranged from 0.5% to 10.8% across 10 studies, although of the 9 studies that were image oriented, eight studies used cranial ultrasound, which is less sensitive in detecting white matter injury, and only one used MRI [16]. Chen et al. [17] found white matter injury in 38 out of 672 (5%) infants using cranial ultrasound and reported a similar incidence in moderate-late preterm infants and very preterm infants [16, 17].

MRI studies undertaken in moderate-late preterm infants at term-equivalent age have also reported a much lower rate of white matter injury than in our study. Walsh et al. [18] found white matter injury in only 5% of 99 moderate-late preterm infants, including 0.5% with a focal punctate lesion, 3.5% with extensive punctate lesions, and 1% with linear lesions. No white matter injury was detected in 50 full-term infants scanned at term-equivalent age [18].

The pathophysiology of white matter injury involves multiple factors, with hypoxic-ischaemic events (e.g. due to placental insufficiency, maternal hypertension, and intrauterine infection) and the associated inflammatory response resulting in damage to the vulnerable maturing oligodendrocytes [17]. Furthermore, excessive release of excitatory neurotransmitters in response to hypoxic-ischaemic events results in further damage to white matter structures [15].

These mechanisms contribute to the observed white matter abnormalities on MRI in very preterm infants, which include focal or diffuse white matter lesions and loss of white matter volume. Regional oedema in response to injury can result in T2 signal hyperintensity [17, 19]. Furthermore, injury resulting in myelin loss disrupts the normal white matter lipid content, resulting in altered T1 and T2/FLAIR signal intensity [17, 19]. The formation of glial scar tissue as a part of the healing process, or gliosis, contributes to altered signal intensity on MRI [17, 19]. The specific MRI findings can vary depending on the individual patient characteristics, underlying cause, timing, duration, and intensity of the underlying insult, as well as individual patient characteristics [17, 19]. As a result, MRI findings may change in appearance over time.

This may be part of the reason why we observed a 10% decrease in the proportion of images in which white matter injury was detected between the early and term-equivalent age scans. These changes may reflect resolution of focal abnormal signal intensity, which is replaced by volume loss over time, making the lesions less apparent on later scans and potentially leading to underreporting of white matter injury in moderate-late preterm infants when MRI is performed closer to term-equivalent age.

However, this 10% decrease in overall incidence of white matter injury obscures a much larger decrease in the severity category of white matter injury between early and term-equivalent scans. More than half of moderate-late preterm infants with early white matter injury categorised as severe were categorised as only moderate severity at term-equivalent age. Similarly, approximately one-quarter of infants categorised as early moderate white matter injury were categorised as mild white matter injury at term-equivalent age, and another quarter had no detectable white matter injury, as did almost three-quarters of infants with early mild white matter injury.

The implications of these findings depend on the clinical importance of white matter injury and its severity in moderate-late preterm infants. If mild white matter injury is found to have no clinical importance for later outcomes, then scanning at term-equivalent age, which is widespread practice in very preterm infants, would miss few clinically important lesions, although a proportion of early moderate and severe white matter injury would be misclassified as mild and moderate. However, if mild white matter injury does have clinical relevance, then, our data suggest that the majority would evade detection on MRI scanning at term-equivalent age.

Strengths of this study include the use of a well-established scoring system, a consistent scanning protocol in a single centre, observers who scored the images independently and were unaware of clinical details including postnatal age, and a large sample size. Limitations include that the study population was drawn from a specific region and was restricted to infants whose parents consented to the study, which may limit the generalisability of the findings to other populations. Furthermore, our study provides no information about how observations may vary with the experience of the observer as well as when assessing images when using different viewing and imaging systems.

We are currently following up this cohort to examine the relationship between early white matter injury severity and long-term developmental outcomes, which will be important for understanding the clinical implications of white matter injury in moderate-late preterm infants. If white matter injury on early MRI is found to be predictive of later outcomes, then identifying white matter injury at an early stage may allow for timely intervention and targeted management strategies to mitigate the potential long-term developmental impairments. Our findings have shown that clinicians could reliably assess the presence and severity of early white matter injury using the UCSF scoring system in moderate-late preterm infants in this context.

## Conclusion

A published scoring system for assessing white matter injury severity on brain MRI in very preterm infants is sufficiently reliable for use by clinicians to describe white matter injury in moderate-to-late preterm infants. White matter injury may be underestimated in these infants when MRI is performed close to term-equivalent age, particularly when the white matter injury is mild.

## Data Availability

De-identified data will be available after publication of key primary papers by the MR DIAMOND and MoPED study groups. Data will be available to researchers who provide a methodologically sound proposal with appropriate ethical and institutional approval, where necessary, and following approval of the proposal by the Data Access Committee at the Liggins Institute. Researchers must sign and adhere to the Data Access Agreement that includes a commitment to using the data only for the specified proposal, to refrain from any attempt to identify individual participants, to store data securely and to destroy or return the data after completion of the project. The Human Health Research Services Platform reserves the right to charge a fee to cover the costs of making data available, if required. Request for access to data can be made to the Human Health Research Services Platform based at the Liggins Institute, University of Auckland (dataservices@auckland.ac.nz).
